# Despotism of Habit

**Published:** 1870-04

**Authors:** 


### DESPOTISM OF HABIT.

THE tyranny, the remorseless exactingness of fostered habits and tastes, ought to make a rational man shudder to contemplate the mental, moral, and physical sufferings which they engender. A correspondent writes: "I think if the city of New York was mine, I would freely give it to get rid of this dreadful habit; it ruins my appetite and makes me miserable. If you can give me anything or do anything to enable me to get rid of the dreadful habit, please let me know." He had been using opium and morphine to ease the pain of chronic rheumatism and promote sleep, and now, in addition to the pain and sleeplessness, he has the "dreadful habit," which, of itself, makes his life a misery. When will the people learn that opium, like alcohol, cures nothing; they are only temporary alleviants, the end being to "bite like a serpent and sting like an adder." The aim of the good and honorable physician is always to eradicate disease, not to plaster it over. What a grand good thing it would be, what a mercy to the poor, to keep in mind this one simple truth: If you are "better" only while you are "taking" any particular medicine, and when you lay it aside, your ailment returns, that medicine is not only doing you no good, but is fixing the malady more firmly in the system, as the cutting off a weed at the level of the ground makes it take a firmer and deeper and stronger root, and you are paying away your hard earned money for the process; and yet we have seen, within a few days, in one of the largest and very best religious newspapers of the day, a richly leaded editorial, speaking in glowing terms of the peculiarly healthful, bracing, and invigorating effects of a good drink of whiskey every morning. Of course he does not call it whiskey; physicians know it to be "made" brandy, with "Bitters" to match; of course, too, he was paid a fee for the insertion as his own of an article, one word of which he never wrote. Countless multitudes have been led into habitual drunkenness by such means, with the result of reputation ruined, estate squandered, wives broken-hearted, and children sent to the poor-house, the brothel, and the penitentiary.

